# Successful treatment of COVID-19-associated collapsing glomerulopathy: 22 months of follow-up 

**DOI:** 10.5414/CNCS111112

**Published:** 2023-07-13

**Authors:** Pulkit Gandhi, Caoimhe Sorcha Dowling, Anjali Satoskar, Ankur Shah

**Affiliations:** 1 Nephrology Department, Rochester General Hospital, Rochester, NY,; 2 Internal Medicine/Nephrology, Lake Erie College Of Osteopathic Medicine, Erie, PA,; 3 Internal Medicine Department Rochester General Hospital, Rochester, NY,; 4 Department of Pathology, Wexner Medical Center, The Ohio State University, Columbus, OH, and; 5 Nephrology Department, Brown University, Providence, RI, USA

**Keywords:** COVID-19, nephrotic range proteinuria, acute kidney injury, FSGS, COVAN, steroids, hematuria

## Abstract

The term COVAN (COVID-19-associated nephropathy) has been used to describe collapsing focal segmental glomerulosclerosis (FSGS) in individuals who have been infected with the SARS-CoV-2. This helps differentiate it from the majority of cases of acute kidney injury in COVID-19 patients, which are typically caused by acute tubular injury. The exact pathophysiology is unclear but is proposed to involve pro-inflammatory cytokines such as type 1 interferons, which are thought to increase expression of the *APOL1* gene in glomerular epithelial cells. This triggers a cascade of inflammatory events that cause damage to the epithelia and underlying podocytes. The treatment of COVAN is centered on general supportive measures including dietary sodium restriction, optimization of hyperlipidemia and hypertension, RAAS blockade, and diuresis for edema. There is limited data to support the use of glucocorticoids in COVAN; however, the mechanism of podocytopathy is similar to that in HIVAN (HIV-associated nephropathy), with high disease burden in those with *APOL1* gene mutation. Based on previous experience, treatment of HIVAN with glucocorticoids is beneficial and safe in selected patients. Here we present a case of COVAN which was successfully treated with glucocorticoids, and at 22-month follow-up patient remained in full remission (proteinuria < 1,000 mg/g) with stable kidney function.

## Case report 

A 58-year-old male presented to the hospital with worsening of dyspnea associated with known COVID-19 infection. He had been in his usual state of health until 10 days before admission when his dyspnea began and he was diagnosed with COVID-19 pneumonia. Other associated symptoms included lower abdominal pain and diarrhea which started on the day of admission. His past medical history included hypertension, hyperlipidemia, prediabetes, obesity, and benign prostatic hyperplasia. Home medications included alfuzosin 10 mg daily, amlodipine 10 mg daily, aspirin 81 mg daily, atorvastatin 40 mg daily, chlorthalidone 25 mg daily, losartan 100 mg daily, and as needed cetirizine 10 mg daily. There were no known drug allergies. Family history was notable for hypertension and malignancy, but no kidney disease. He denied any tobacco use. 

On examination, temperature was 38.3 °C (101 °F), blood pressure 146/92 mmHg, heart rate 65 beats per minute, respiratory rate 20 breaths per minute, and oxygen saturation 97% on room air. The body mass index (weight in kilograms divided by the square of the height in meters) was 35.4. Cardiopulmonary exam revealed a normal rate and rhythm without adventitious heart sounds. The mucous membranes were moist. Pitting edema was noted in the bilateral lower extremities. The remainder of the physical examination was unremarkable. Presentation labs were notable for serum sodium 136 mmol/L, potassium 3.4 mmol/L, chloride 100 mEq/L, CO_2_ 31 me/L, blood urea nitrogen 44 mg/dL, creatinine 2.6 mg/dL, calcium 8.1 mg/dL, and albumin 3.2 g/dL. Labs obtained 1 day prior to admission were notable for serum creatinine of 2.35 mg/dL with a prior baseline of 1.1 mg/dL last checked 5 months before presentation. 

**Figure 1.**
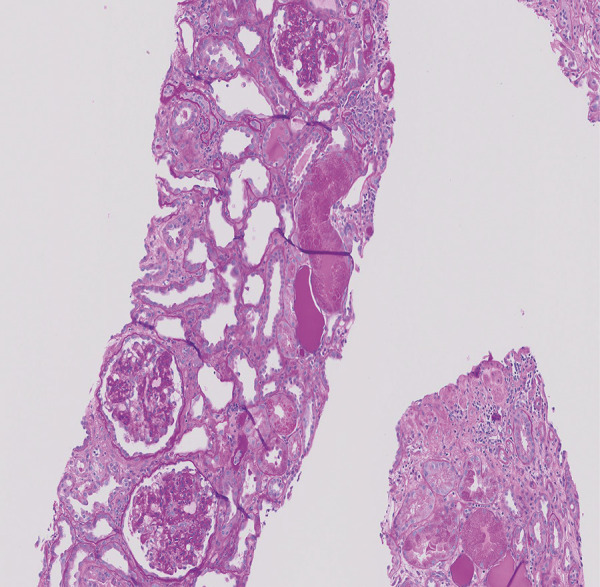
Kidney biopsy light microscopy showing collapsed glomeruli.

**Figure 2. Figure2:**
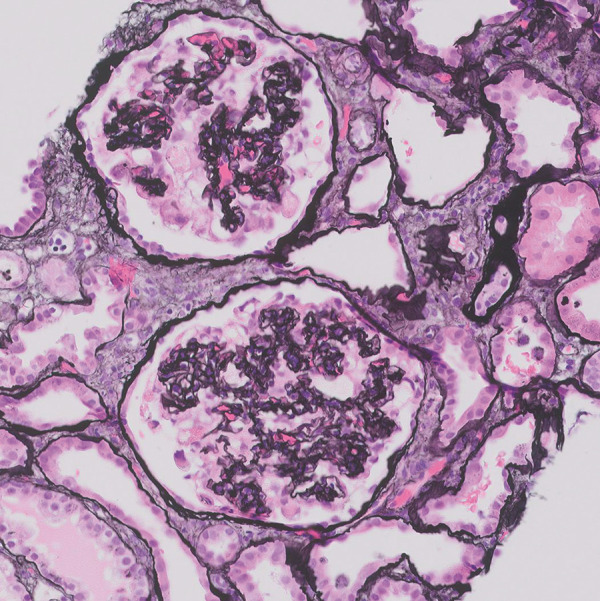
Jones silver stain better highlights the collapsed glomerular capillary loops without surrounding podocytes.

Initial management was focused on acute hypoxic respiratory failure, and the patient was treated with supplemental oxygen, remdesivir, and dexamethasone. The hospitalization was complicated by further worsening of his acute kidney injury with serum creatinine rising from 2.6 mg/dL at the time of admission to 5.2 mg/dL over a 7-day period. Further lab work showed severe hypoalbuminemia (1.8 g/dL), and the new-onset edema of the bilateral lower extremities noted on physical exam worsened. Urinalysis was significant for hematuria with 6 – 10 RBC/HPF and > 300  mg/dL proteinuria. Computerized tomography of the abdomen demonstrated no hydronephrosis or nephrolithiasis. 24-hour urine protein was measured at 35,920 mg. Immunological work-up came back negative for ANA, ANCA, anti-dsDNA, and showed normal levels of C3 and C4. He then underwent a kidney biopsy. The light microscopy showed widely collapsed glomeruli with enlarged protein-containing podocytes (Figure 1), flattened tubular epithelia with large hyaline casts, and mild patchy interstitial inflammation in 100% of glomeruli in the sample. Interstitial fibrosis and tubular atrophy was estimated at less than 20% of the renal cortex in the biopsy specimen. Immunofluorescence showed no evidence of glomerular immune complex deposits. Electron microscopy could not be performed, as stained sections contained no glomeruli. Pathology findings were consistent with severe collapsing glomerulopathy, most likely secondary to COVID-19 infection.[Fig Figure2]

At the time, there was no strong data regarding the approach to treatment of COVID-19-associated collapsing glomerulopathy (COVAN). However, given the severity of proteinuria and acuity of onset, the decision was made to commence treatment with high-dose prednisone. For the first 12 weeks, the patient took 60 mg of prednisone daily. After that period, 24-hour urine collection was repeated and showed an almost 10-fold reduction in proteinuria from 35,920 to 3,691 mg. Serum albumin was also repeated and had improved to 3,691 mg measured by 24-hour urine. In addition, serum creatinine had improved to 3.2 mg/dL. A slowly tapering course of prednisone was completed over the following weeks. At 22 months, repeat testing showed serum creatinine 2.6 mg/dL, albumin 4.5 g/L, and urine protein-to-creatinine ratio of 553 mg/g. 

## Discussion 

Collapsing glomerulopathy, also known as collapsing focal segmental glomerulosclerosis (FSGS), is debated to be either a subtype of FSGS or its own entity. It has recently been recognized as an important cause of end-stage kidney disease (ESKD) [16]. It is characterized by a pattern of renal injury including tubular cystic dilation, proteinaceous casts, hypertrophy and hyperplasia of glomerular epithelia, and collapse of the glomerulus away from the retracted basement membrane [6]. Collapsing glomerulopathy has historically been associated with HIV infection and has been called HIV-associated nephropathy (HIVAN) [1]. The pathogenesis of HIVAN involves direct infection of kidney epithelial cells with a subsequent expression of HIV in genetically susceptible host. This explains the high incidence of HIVAN in people of African-American ethnicity who have higher chance of carrying *APOL1* gene mutation. More recently, non-HIV viruses have been implicated in its pathogenesis, and these include parvovirus B19, cytomegalovirus, human T-cell lymphotropic virus-1, hepatitis C [12], and COVID-19 [2, 13]. Some parasitic infections like filariasis, leishmaniasis, and loa loa have also been implicated in addition to the viruses mentioned here.. The exact pathophysiology is unclear but is proposed to involve pro-inflammatory cytokines such as type 1 interferons, which are thought to increase expression of the *APOL1* gene in glomerular epithelial cells. This triggers a cascade of inflammatory events that cause damage to the epithelia and underlying podocytes [3]. It has also been proposed that the COVID-19 virus may have direct access to renal parenchyma via the receptor activity of angiotensin-converting enzyme 2 [4], although evidence for this has been inconsistent and does not allow for unequivocal confirmation of the hypothesis [5]. 

The role of genetics in renal disease has been widely studied. Specifically, the *APOL1* gene and its genetic variants have been shown to have a strong association with renal disease [3]. The protein product of *APOL1* – apolipoprotein 1 – has multiple functions including trypanosome lysis, lipid metabolism, cellular senescence, and autophagocytosis [7]. Two variant alleles with increased prevalence in sub-Saharan Africa, G1 and G2, have been linked with a higher risk of renal disease and are thought to provide a protective effect against trypanosomiasis [3]. More recent research has demonstrated that G1 and G2 variant alleles may increase the risk of COVID-19-associated collapsing glomerulopathy [4, 8, 9], including in the case above where genetic testing revealed homozygous *APOL1* mutation (G1 allele). The role of apolipoprotein 1 in renal disease is unclear, but may involve lysosomal dysfunction, increased endoplasmic reticulum stress, and osmotic dysregulation at a cellular level [10]. 

Prognosis of COVAN unfortunately remains poor [14, 15]. As per recently published data on patients affected with COVAN, there is an overall poor prognosis, with most patients developing advanced chronic kidney disease and one-third of patients with ESKD or death at < 1 year [14]. 

The treatment of COVAN is typically supportive with interventions including dietary sodium restriction of 2.3 g/day, optimization of hyperlipidemia and hypertension, blockade of the renin-angiotensin-aldosterone system, sodium/glucose cotransporter-2 inhibition, and maintenance of euvolemia. There is limited data to support the use of glucocorticoids in COVAN; however, the mechanism of podocytopathy is postulated to be similar to that in HIVAN. Based on previous experience, treatment of HIVAN with glucocorticoids is beneficial and safe in selected patients [11]. Data to date shows poor outcomes in patients with COVID-19-associated collapsing glomerulopathy, with most patients needing hemodialysis [9]. 

Our case demonstrates that corticosteroids should be considered an option in the treatment of COVAN. Having said that, there is a possibility that this patient might have recovered without the use of steroids, but we did wait for 1 month post biopsy before attempting the use of high-dose prednisone. Further research is needed to identify the optimal treatment approach. 

## Funding 

This research did not receive any specific grant from funding agencies in the public, commercial, or not-for-profit sectors. 

## Conflict of interest 

The authors declare that they have no conflict of interest.
